# *Asplenium pseudocapillipes* (Aspleniaceae), a New Fern Species from South Korea

**DOI:** 10.3390/plants11223089

**Published:** 2022-11-14

**Authors:** Sang Hee Park, Jung Sung Kim, Hyoung Tae Kim

**Affiliations:** 1 Department of Forest Science, Chungbuk National University, Chungdae-Ro 1, Seowon-Gu, Cheongju 28644, Chungbuk, Korea; 2 Department of Ecological and Environmental System, Kyungpook National University, Gyeongsang-Daero, Sangju-Si 37224, Gyeongsangbuk-Do, Korea

**Keywords:** *Asplenium pseudocapillipes*, new species, allotetraploid, *Asplenium tenuicaule*, *Asplenium capillipes*

## Abstract

A new allotetraploid species of the genus *Asplenium*, *A. pseudocapillipes*, originated from the hybridization between *A. capillipes* and *A. tenuicaule*, has been newly discovered in two limestone areas of South Korea. A molecular phylogenetic analysis using one chloroplast region (*rbcL*) and three single- or low-copy nuclear regions (*AK1*, *gapCp*, *pgiC*) and a cytological analysis, including genome size measurements, were conducted to characterize this new species. From these results, the maternal origin of *A. pseudocapillipes* was confirmed to be *A. capillipes*, which has never been reported in Korea. All three nuclear data showed that this new species had genotypes of both *A. capillipes* and *A. tenuicaule*. The quantitative characteristics of the leaves showed values intermediate between the two parental species. The absence of gemma accorded with its paternal origin from *A. tenuicaule*, and 32 spores per sporangium accorded with its maternal origin from *A. capillipes*. Although *A. pseudocapillipes* has 32 spores per sporangium, it is considered to be a sexually reproducing, not an apomitic, fern.

## 1. Introduction

Polyploidization is considered an important factor in plant evolution [1,2,3,4]. Polyploids can be generated by autopolyploidization and allopolyploidization. Autopolyploidization occurs by doubling the genome within a species, but allopolyploidization occurs by interspecific hybridization followed by genome doubling [5]. In ferns, the ratio of speciation events related to polyploidy is higher (31%) than that in angiosperms (15%) [6]. Because of the frequent polyploidization and hybridization that occurs in ferns, they can sometimes experience reticulate evolution [7]. As a result, it can form a species complex that has morphological continuity between species [8]. To examine this morphological complexity in related taxa, many researchers have attempted to apply genome size measurement or low-copy nuclear DNA markers for genotyping analysis in ferns [9,10,11,12,13,14,15,16]. Through these attempts, new species, that were previously unknown and unrecognized due to their morphological similarities, have actually been identified [9,14,17].

Comprising approximately 700 species, *Asplenium* L. (Aspleniaceae) is a species-rich fern genus [18,19]. Members of *Asplenium* show various ploidy levels [20,21] and form a species complex comprising basic diploids and many auto- and allopolyploids by frequent reticulate evolution [9,10,14,15]. Moreover, it is possible to produce sterile hybrids between species with different ploidy levels or between homoploids in the same complex [15]. As the presence of numerous morphologically intermediate species in the genus *Asplenium* make it more difficult to distinguish one from another, researchers have attempted to solve this problem using various methods, including cytological and molecular methods [9,10,15,16,22,23].

To investigate *Asplenium* species, we collected plant samples from various habitats in Korea. Among them, we found plants that had been collected from the limestone area and identified as *A. tenuicaule* Hayata in the field based on their morphological characteristics. However, they were different from typical *A. tenuicaule* because the plants had an acute segment that is not generally observed in this taxon. This newly observed *Asplenium* sp. was more similar to *A. capillipes* Makino, except for the absence of gemma in the acroscopic axil.

In this study, cytological and molecular phylogenetic analyses were conducted to investigate the identity of this enigmatic species. Our goals in this paper were (1) to verify whether the newly found *Asplenium* sp. originated from hybridization; (2) to identify the parent species if it was a hybrid taxon; and (3) to describe it in detail if it was recognized as a new species.

## 2. Results

### 2.1. Chloroplast Phylogeny Based on rbcl

**Figure 1:**
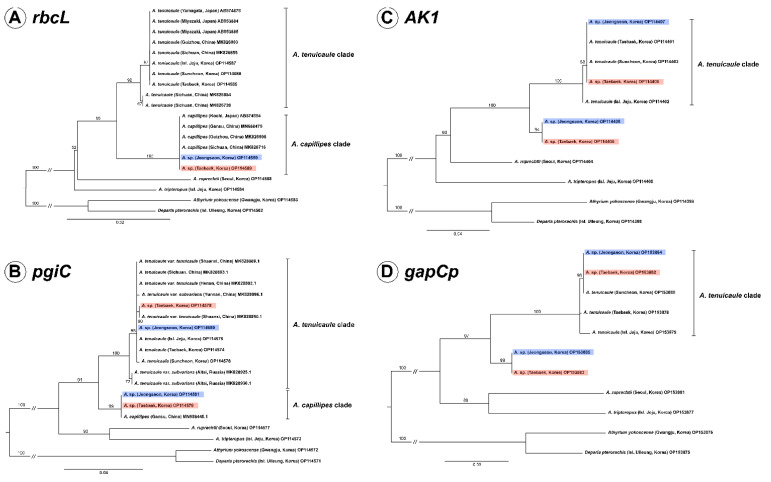
Maximum likelihood phylogeny based on plastid *rbcL* (**A**) and three nuclear markers, *pgiC* (**B**), *AK1* (**C**), and *gapCp* (**D**) of *Asplenium* species and related species. *Asplenium* sp. is marked in red (Collection Site A) and blue (Collection Site B) boxes. Maximum likelihood bootstrap values are shown above the branches. The scale bar indicates a branch length corresponding to substitutions per site.

We determined the *rbcL* sequences of the new taxon *Asplenium* sp. found in Korea and its related species in this study and compared them together with the sequences of *A. capillipes* and *A. tenuicaule* reported from Japanese and Chinese samples. From the result, it was confirmed that the sequence of new taxon was identical to those of *A. capillipes* of Japan and China. The aligned sequence length of *rbcL* was 1272 bp with 1090 conserved sites and 63 parsimony-informative sites. The phylogenetic analysis showed that two individuals of *Asplenium* sp. were nested in the *A. capillipes* clade comprising Chinese and Japanese specimens with 100% ML bootstrap support, and the *A. capillipes* clade was identified as being sister to the *A. tenuicaule* clade (Figure 1A).

### 2.2. Nuclear Phylogenies Based on pgiC, AK1, and gapCp

After alignment, the lengths of the nuclear genes *pgiC*, *AK1*, and *gapCp* were found to be 694 bp, 866 bp, and 1221 bp, respectively. In *pgiC*, one copy of *Asplenium* sp. from each population (non-T type) was completely identical to the *pgiC* of Chinese *A. capillipes*, except for the 1 bp deletion of poly T in the Jeongseon population (CBNU2020-0171A). The other copy of *Asplenium* sp. in each population was almost identical to *A. tenuicaule,* therefore, we designated this copy as T type (Supplementary Figure S1). In the phylogeny based on *pgiC*, the T types of *Asplenium* sp. were nested in the *A. tenuicaule* clade, and the non-T types were positioned in the *A. capillipes* clade with an ML bootstrap support >99% (Figure 1B). 

In the cases of *AK1* and *gapCp,* each population of *Asplenium* sp. had two copies. One copy was identical to the sequence of *A. tenuicaule*, but the other was obviously different. The phylogeny based on *AK1* and *gapCp* showed that the T types of *Asplenium* sp. and *A. tenuicaule* were also strongly supported as being monophyletic, and the non-T types of *Asplenium* sp. formed an independent clade with an ML bootstrap support >94% (Figure 1C,D). 

### 2.3. Ploidy Analysis and Spore Observation

**Figure 2:**
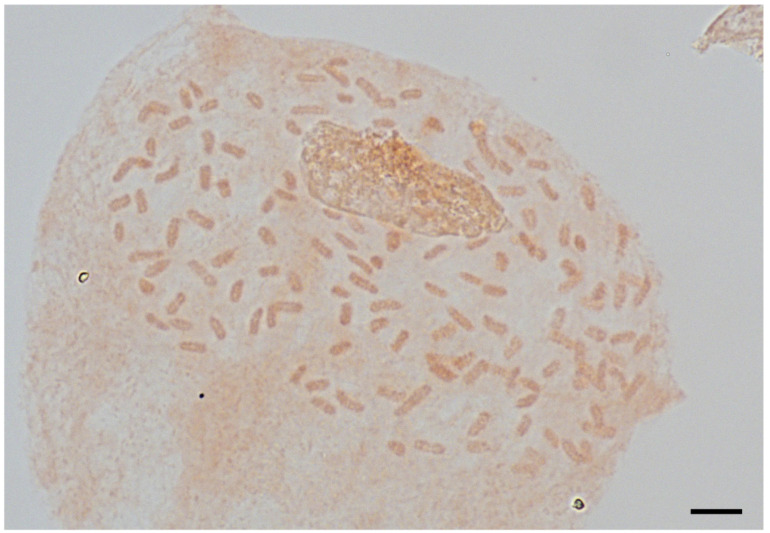
The mitotic chromosomes of *Asplenium* sp., 2n = 144. Microscopic photo. Scale bar = 10 μm.

**Figure 3:**
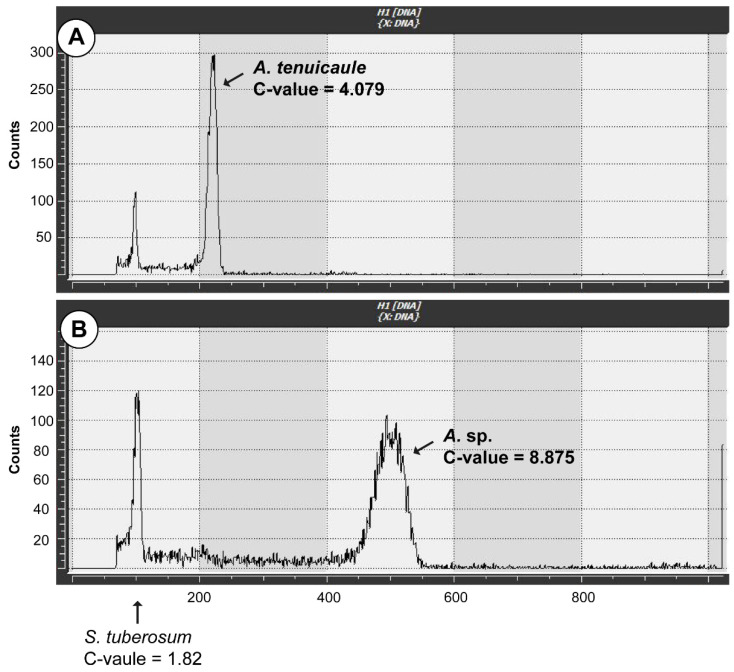
Results of genome size measurement using a flow cytometer. (**A**) *Asplenium tenuicaule*, (**B**) *Asplenium* sp.

The mitotic chromosome number of *Asplenium* sp. was 2n = 144. Therefore, it was identified as being tetraploid (Figure 2). The genome size of *Asplenium* sp. with 16 individuals and *A. tenuicaule* with 4 individuals were measured and the mean C-values were 8.875 ± 0.06 pg and 4.079 ± 0.05 pg, respectively (Figure 3).

**Figure 4:**
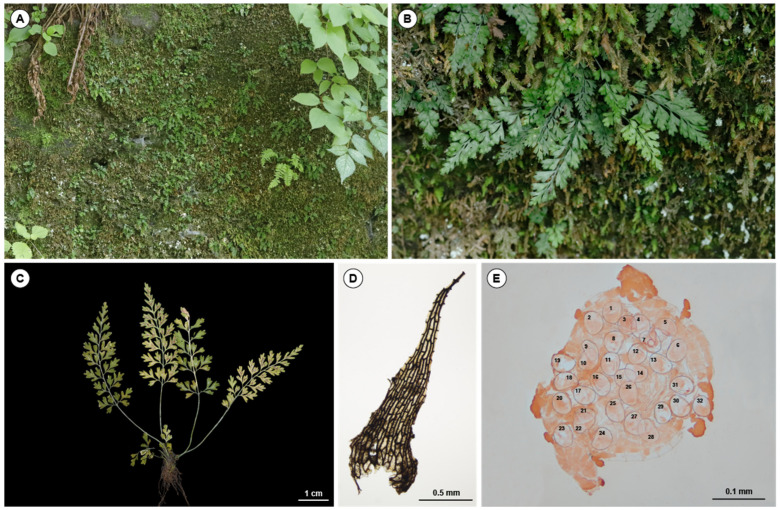
*Asplenium pseudocapillipes* sp. nov. (**A**,**B**) Habitat. (**C**) Plant. (**D**) Rhizome scale. (**E**) Thirty-two spores per sporangium.

In addition, 32 spores were observed per sporangium (Figure 4E), with a mean size of 52.94 ± 3.02 μm ranging 46–59 μm, and they successfully germinated (Supplementary Figure S2).

Etymology: the species resembles *A. capillipes*.

From the results, we conclude *Asplenium* sp. should be treated as the new allotetraploid species *Asplenium pseudocapillipes* (Figure 4), which is described as follows.

### 2.4. Taxonomic Treatment

***Asplenium pseudocapillipes*** S. H. Park, J. S. Kim & H. T. Kim, **sp. nov.** Type: South Korea, Taebaek-si, Hasami-dong, Deokhang Mountain, 770 m alt., 30 April 2020, (holotype CBNU2020-0106)

Diagnosis: *Asplenium pseudocapillipes* is allotetraploid, originating as the hybrid between *A. capillipes* and *A. tenuicaule,* and generally shows the intermediate form of both species (Table 1). This species has an acute segment apex and 32 spores per sporangium, which are similar to those of *A. capillipes*, but a gemma of the acroscopic axil does not form as in *A. tenuicaule*.

Plants are evergreen and epilithic. Plants (1.5-3-9(-12) cm tall. Rhizome shortly erect, apex scaly; scales dark brown to black, triangular to narrowly triangular. Fronds caespitose, herbaceous, green, subglabrous; stipe slender, sulcate adaxially, (0.3–)0.5–3(–5) cm; lamina triangular to narrowly triangular, (0.8-)1.5-6(-7) × (0.5-)0.7-2.5(-3) cm, 2 or almost three-pinnate, apex acute to acuminate, without gemma; pinnae (3-)4-9(-11) pairs, alternate or opposite, stalk slender, basal pinnae not reduced (or often slightly reduced), broadly ovate to triangular, 3-11(-15) × 2.5-7(-9) mm in basal pinna, pinnate to two-pinnatifid; ultimate segments (1-)1.5-3(-5) × 1-2.5(-3.5) mm, base cuneate, margin entire, apex mucronate to acute. Sori, one (or two) per ultimate segment or pinnule, basal to median on subtending vein, linear to narrowly oblong, (0.8-)1-1.8(-2.5) mm; indusia whitish to whitish-yellow, membranous, margins entire, opening toward costa, persistent. Spores 32 per sporangium, longer diameter averaging 52.94 μm, ranging 46-59 μm, excluding perispores. Tetraploid, 2n = 144.

Distribution and habitat: Two populations of *A. pseudocapillipes* have been reported from the limestone areas of Korea in Gangwon-do, Jeongsun-gun, and Taebaek-si. It usually grows by forming clusters on mossy rocks under moist forests.

## 3. Discussion

### 3.1. Discovering New Allotetraploid in Genus Asplenium

In this study, we found a new allotetraploid species, *A. pseudocapillipes*, from two populations in limestone areas of Korea. It was different from the hybrid species *Asplenium* × *capillicaule* Fraser-Jenk. from Nepal [25] and Japan [26] between *A. capillipes* and *A. tenuicaule* because *Asplenium* × *capillicaule* is known to have a gemma in acroscopic axils, which is similar to that of *A. capillipes*. 

The *A. pseudocapillipes* found in Korea had two distinct copies of all three nuclear genes tested in the present study. The T types or non-T types (*pgiC*) of *A. pseudocapillipes* formed a clade with the counterparts of *A. tenuicaule* and *A. capillipes*, respectively. Maternally inherited *rbcL* was shown to be the maternal parent of *A. pseudocapillipes*. Based on the number of chromosomes, genome size, and fertile spores of *A. pseudocapillipes*, it is an allotetraploid between *A. capillipes* and *A. tenuicaule* because both parent species are diploids [18,24,26], excluding the tetraploid of *A. capillipes* in the Himalayas [27].

**Figure 5:**
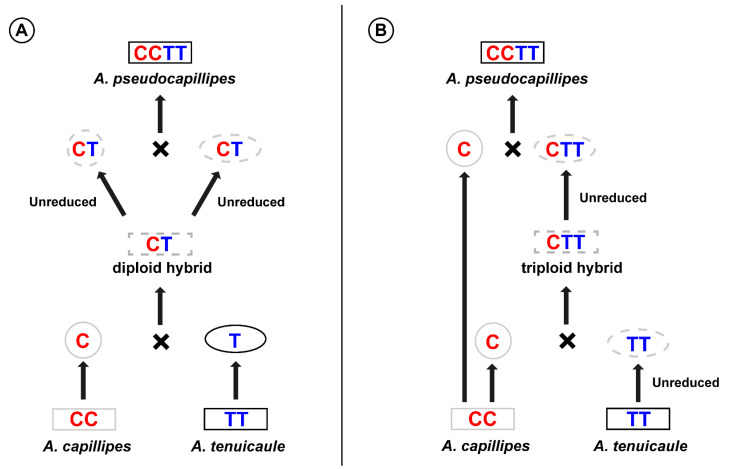
Two scenarios for the origin of *A. pseudocapillipes*. (**A**) Direct allopolyploidization via diploid hybrid. (**B**) Allotetraploidization via triploid bridge. Boxes represent each taxon and circles indicate gametes. Solid and dashed lines indicate known and putative taxa or gametes, respectively. The colors of the lines indicate whether it is native to Korea: black, native; gray, not native or unreported.

Allotetraploid *A. pseudocapillipes* may originate by two unreduced gametes of a homoploid hybrid (Figure 5A) or by a triploid bridge (Figure 5B). Because we have not yet found any sterile hybrids of diploid or triploid *A. pseudocapillipes*, it is not easy to establish a speciation model for this allotetraploid. The paternal species *A. tenuicaule* grows closely with *A. pseudocapillipes* in Korea, whereas the maternal species *A. capillipes* has not been reported in Korea yet. This implies that *A. pseudocapillipes* moved into Korea after speciation or that *A. capillipes* became extinct in Korea after speciation. However, *A. capillipes* is small and grows among moss with a high morphological similarity to *A. tenuicaule* [18] and *A. pseudocapillipes*. Therefore, this species may have been easily overlooked or was identified as *A. tenuicaule* even though we did not find any *A. capillipes,* which was identical to our original description, from herbarium specimens survey. If *A. capillipes* grew closely with *A. tenuicaule* and hybridization and polyploidization recently occurred, the relatively low genetic diversity of nuclear sequences between parents and offspring is to be expected.

In this study, we compared new allotetraploids to their parental species using morphological characters and cytological and molecular analyses. Further studies including gametogenesis and sporogenesis are required to deepen our understanding of this species.

### 3.2. Reproductive Mode of Asplenium Pseudocapillipes

During sporogenesis, most sexual reproductive leptosporangiate ferns produce 16 spore mother cells through four premeiotic mitoses and a total of 64 haploid spores per sporangium through meiosis [28]. In contrast, apomictic ferns have 32 spores with the same number of chromosomes as the parent sporophyte either by premeiotic endomitosis [29,30] or meiotic first division restitution [31]. Therefore, 32 spores per sporangium were considered to be presumptive evidence of apomictic ferns [29,30,32]. However, some species have been confirmed to be sexually reproductive ferns, even though they have 32 spores, such as Lindsaeaceae species [33] and *Cystodium sorbifolium* [34], or 16 spores, such as *Alsophila* species [35]. Lin, Kato, and Iwatsuki [34] deduced that the formation of eight spore mother cells was due to the reduction in premeiotic mitosis divisions from four to three and referred to these species types as “32-spored sexual type” or “lindsaeoid type”.

In *Asplenium*, there are some species with 32 spores, such as *A. monodon* [36], *A. monanthes*, *A. palmeri* [10], *A. heterochroum*, *A. resilens* [37], and *A. cheilosorum* [38], which are similar to *A. pseudocapillipes* found in the present study. Therefore, this poses the question of whether *A. pseudocapillipes* is agamosporous. To answer this question, we need to focus on the parents. *A. capillipes* is the maternal parent of *A. pseudocapillipes*, and is considered to be an apogamous species because it has 32 spores per sporangium [39]. However, half of the parental chromosomes in meiosis I, the normal meiotic division, and eight spore mother cells imply that this species is sexual in reproduction [24]. In contrast, *A. tenuicaule*, the paternal parent of *A. pseudocapillipes*, normally produces 64 spores in the sporangium. Therefore, it was suggested that the feature of “32-spored sexual type” found in *A. pseudocapillipes* was likely to be inherited from its paternal parent *A. capillipes*.

## 4. Materials and Methods

### 4.1. Plant Materials and Observation of Morphological Characteristics 

**Figure 6:**
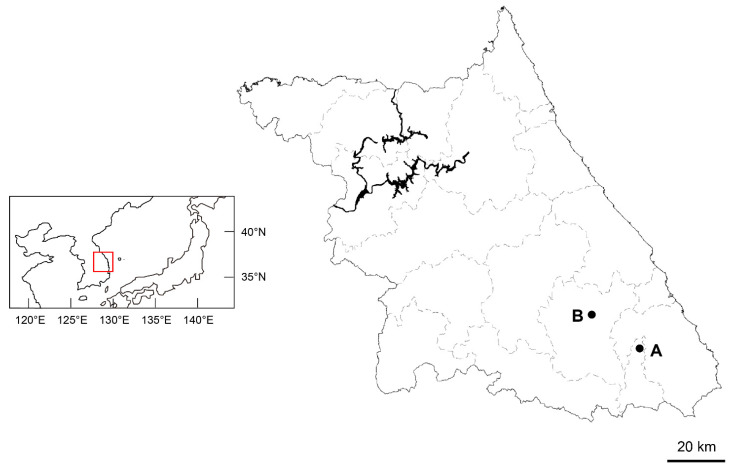
Collection sites for *Asplenium* sp. A: Taebaek-si. B: Jeongsun-gun.

Except for the populations mentioned in the introduction, we found another population of *Asplenium* sp. and *A. tenuicaule* within a 10 m radius in the limestone areas of Gangwon-do, Korea (Figure 6). Living samples were collected from each population and transplanted into the greenhouse at Chungbuk National University (Cheongju, Chungbuk, Korea), and voucher specimens (Table 2) were deposited in the herbarium of the Chungbuk National University (CBNU). One individual from each of the two populations of the new taxon and three diploid *Asplenium* species (*A. tenuicaule*, *A. ruprechtii* Kurata, and *A. tripteropus* Nakai) were used for the molecular analysis. Two diploid Athyriaceae species (*Athyrium yokoscense* (Franch. & Sav.) Christ, and *Deparia pterorachis* (Christ) M. Kato were used as outgroups for the phylogenetic analysis (Table 2). 

Rhizome scales and spores were observed using a light microscope (Olympus BX50, Tokyo, Japan). A total of 35 spores were randomly sampled under a light microscope (Olympus BX50) to measure the spore size based on the length of the long axis, excluding the perispore. 

### 4.2. Chromosome Counting and Measurement of the Genome Size

For the observation of the mitotic chromosomes, the root tips were pretreated using 2 mM 8-hydroxyquinoline solution for 2 h and then fixed in Carnoy’s solution for 12 h. The fixed root tips were washed with 70% ethanol, macerated in 1 N HCl for 5 min, and then stained using the squashed method with 1% aceto-orcein. The slides were examined at 1000× magnification and then captured using a light microscope (Olympus BX50).

### 4.3. DNA Extraction, PCR Amplification, and Cloning

Genomic DNA was extracted from the leaves dried with silica gel using a DNeasy Plant Mini Kit (Qiagen, Hilden, Germany) following the manufacturer’s protocol. Based on maternally inherited chloroplast genomes and biparentally inherited nuclear genomes in the genus *Asplenium*, chloroplast *rbcL* and nuclear *pgiC* regions were amplified to clarify the parent species, given that the sequences of two putative parent species, namely *A. capillipes* and *A. tenuicaule*, have been previously reported (Figure 1A,B). Nuclear *AK1* and *gapCp* regions were amplified to confirm the hybrid origin of the new taxon of *Asplenium* sp. Primer sets for *rbcL*, *1FN* [40], and *1361R* [41], *AK1*, *AK_4F*, and *AK_R2* [42], *gapCp*, *ESGAPCP8F1*, and *ESGAPCP11R1* [43], and *pgiC*, *14F*, and *16R* [44] were used for the PCR amplification. However, given that the *pgiC* amplification was not successful for *Asplenium* sp., the new primer set, *14F2* (5′-GAGTGTTTGGAATGTTTCCTTC-3′) and *16R3* (5′-GAGGAATGCCATCTATTGAA-3′), was newly designed for this study. The reaction mixture comprised 10 μL of AccuPower^®^ PCR Premix (Bioneer, Daejeon, Korea), 1 μL of DNA, 1 μL of each primer (10 pM), and distilled water to a total volume of 20 μL. The PCR conditions were as follows: a total of 5 min denaturation at 95 °C, followed by 30 cycles at 95 °C for 45 s, 53 °C for 20 s, 72 °C for 60 s, followed by a final extension step at 72 °C for 10 min. Except for nuclear markers of *Asplenium* sp., the PCR products were purified using Expin™ PCR SV (GeneAll, Seoul, Korea) and sequenced using the AB1 3730xl System (Macrogen, Seoul, Korea). The PCR products of nuclear markers of *Asplenium* sp. were purified using the PureLink™ PCR purification kit (Invitrogen, Waltham, MA, USA) to remove primer dimers and small fragments of less than 300 bp. To obtain accurate sequences of nuclear markers, cloning was performed using the TOPO™ TA Cloning™ Kit (Invitrogen) following the manufacturer’s protocol. At least 10 colonies were randomly selected from each plate and grown in liquid medium. Plasmids were extracted using Exprep™ Plasmid SV (GeneAll) and sequenced with a universal M13 primer using the AB1 3730xl System (Macrogen). 

### 4.4. Sequence Alignments and Phylogenetic Analyses

Each of the cloned nuclear genes was divided into two types, and a consensus sequence of each type was generated with a threshold of 50% in Geneious Prime software (ver. 2022.0.2) [45]. One chloroplast and three nuclear genes were aligned using MAFFT [46] and MUSCLE [47], respectively. For the phylogenetic analysis, the best-fit model of nucleotide substitution for each dataset was determined using ModelFinder [48] and a maximum likelihood analysis was performed using IQ-Tree [49] with 1000 ultrafast bootstraps [50].

### 4.5. Genome Size Measurement

The genome sizes of *Asplenium* sp. and *A. tenuicaule* were measured using a CyFlow^®^ Ploidy Analyzer (Sysmex-Partec, Munster, Germany). Tetraploid *Solanum tuberosum* L., 1C = 1.82 pg [51], was used as the internal standard. Young fresh leaf tissues of the two *Asplenium* species and *S*. *tuberosum* were collected from living samples. They were washed using distilled water and chopped with razor blades in 500 μL of CyStain UV Precise P nuclei isolation buffer (Sysmex-Partec). After 10 min of incubation on ice, the suspension was then filtered through a nonsterile CellTrics^®^ 30 μm filter and stained using 2 mL of UV Precise P staining buffer (Sysmex-Partec) containing DAPI. The stained suspension was loaded onto a flow cytometer, and the genome size was measured.

## Figures and Tables

**Table 1:** A comparison of *A. pseudocapillipes* and two parental species. Measurements of *A. capillipes* and *A. tenuicaule* were referred to in the Flora of China [18]. Asterisk (*) is a measurement of the spore length according to Ichihara [24].

	*A. capillipes*	*A. pseudocapillipes*	*A. tenuicaule*
**Plant height (cm)**	3–8	3–9	6–15
**Stipe (cm)**	0.3–2.5	0.5–3	1.5–5
**Lamina (cm)**	1.5–6 × 0.5–2.5	1.5–6 × 0.7–2.5	4–9 × 1.2–3
**Number of pinnae**	5–7	4–9	7–10
**Spore length (μm)**	38–42 45.7 *	46–59	28–32 29.1 *
**Ploidy**	2×, 4×	4×	2×
**Number of spores per sporangium**	32	32	64
**Gemma on rachis**	Present	Absent	Absent

**Table 2:** List of plant materials used for DNA analysis in this study.

Voucher	Locality	Accession			
		*rbcL*	*AK1*	*gapCp*	*pgiC*
***Asplenium* sp.**					
CBNU2021-0084-A	Korea: Gangwon-do, Taebaek-si	OP114589	OP114405 P114406	OP153882 P153883	OP114578 P114579
CBNU2020-0171-A	Korea: Gangwon-do, Jeongsun-gun	OP114590	OP114407 P114408	OP153884 P153885	OP114580 P114581
***Asplenium tenuicaule***					
CBNU2020-0096-E	Korea: Jeollabuk-do, Suncheon-si	OP114586	OP114403	OP153880	OP114576
CBNU2020-0157-D	Korea: Jeju Island, Jeju-si	OP114587	OP114402	OP153879	OP114575
CBNU2021-0086-D	Korea: Gangwon-do, Taebaek-si	OP114585	OP114401	OP153878	OP114574
***Asplenium ruprechtii***					
CBNU2020-0180-A	Korea: Seoul, Gangbuk-gu	OP114588	OP114404	OP153881	OP114577
***Asplenium tripteropus***					
CBNU2020-0028-A	Korea: Jeju Island, Jeju-si	OP114584	OP114400	OP153877	OP114573
***Athyrium yokoscense***(outgroup)					
CBNU2021-0104-A	Korea: Jeollanam-do, Gwangju-si	OP114583	OP114399	OP153876	OP114572
***Deparia pterorachis*** (outgroup)					
CBNU2021-0017-A	Korea: Gyeongsangbuk-do, Ulleung Island	OP114582	OP114398	OP153875	OP114571

## Data Availability

Data generated in the present study were deposited in the NCBI database and available from the website (accessed on 1 December 2022, https://www.ncbi.nlm.nih.gov/nuccore/).

## Supplementary Materials

The following supporting information can be downloaded at: https://www.mdpi.com/article/10.3390/plants11223089/s1, Figure S1: Comparison of pgiC gene sequence after editing the common Indels and outgroups in the original alignment among the *Asplenium* sp. and its putative parental species; Figure S2: Germinated spores of *A. pseudocapillipes* in the 1/2 MS medium 45 days after sowing. Scale bar = 0.1 mm.
