# Supplementary material for: Asplenium pseudocapillipes (Aspleniaceae), a New Fern Species from South Korea
Source: Plants (Basel). 2022 Nov 14;11(22):3089. doi: 10.3390/plants11223089 (PMC9696321; doi:10.3390/plants11223089)
Supplement: Supplementary file 1 [file plants-1972835-supplementary.pdf › plants-1972835-supplementary.pdf]

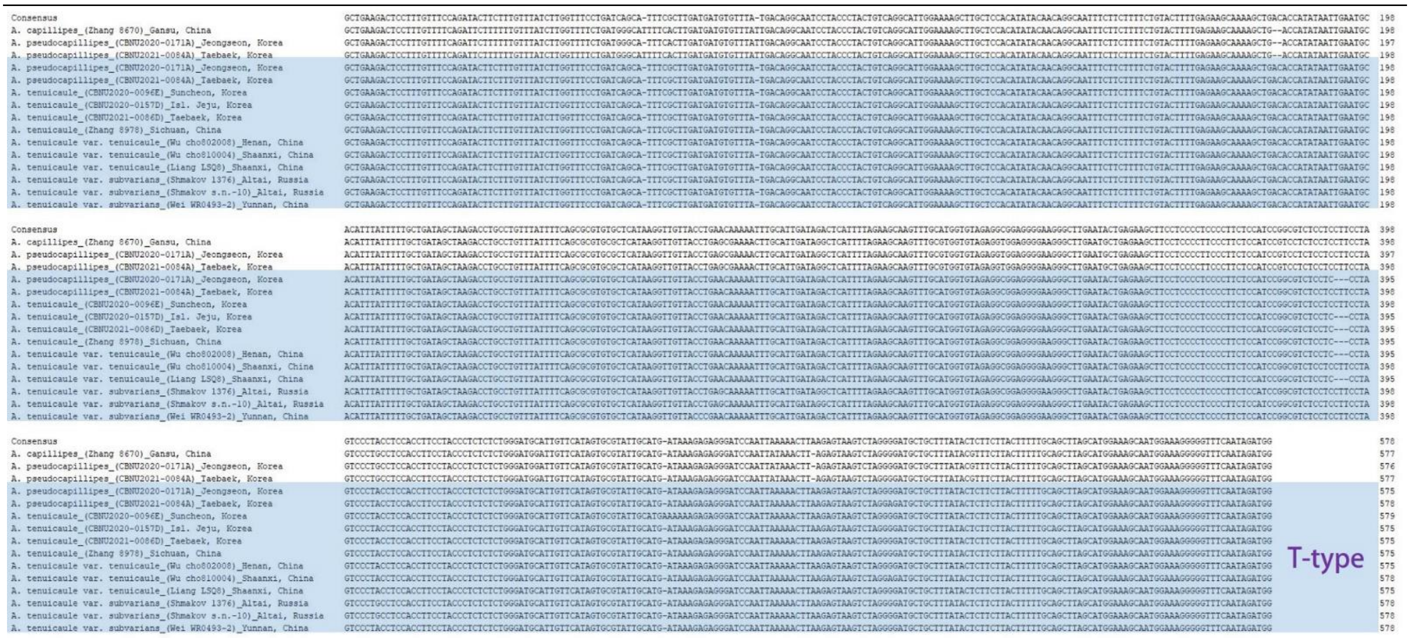

Supplementary Figure S1. Comparison of *pgiC* gene sequence after editing the common Indels and outgroups in the original alignment among the *Asplenium* sp. and its putative parental species.

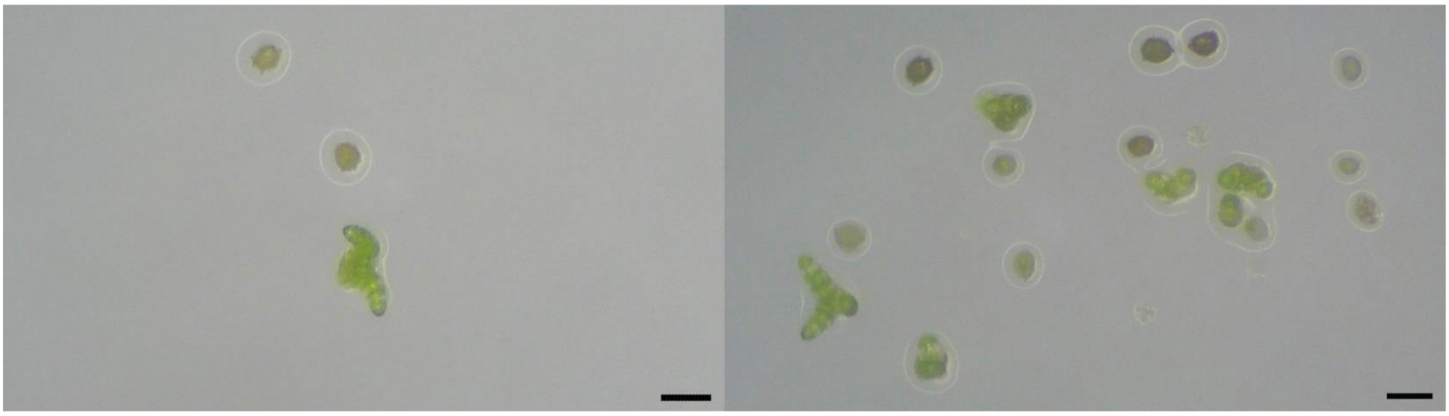

Supplementary Figure S2. Germinated spores of *A. pseudocapillipes* in the 1/2 MS medium 45 days after sowing. Scale bar = 0.1 mm.
